# Recommendations for Use of Video Directly Observed Therapy During Tuberculosis Treatment — United States, 2023

**DOI:** 10.15585/mmwr.mm7212a4

**Published:** 2023-03-24

**Authors:** Joan M. Mangan, Rachel S. Woodruff, Carla A. Winston, Scott A. Nabity, Maryam B. Haddad, Meredith G. Dixon, Farah M. Parvez, Carissa Sera-Josef, LaTweika A. T. Salmon-Trejo, Chee Kin Lam

**Affiliations:** 1 Division of Tuberculosis Elimination, National Center for HIV, Viral Hepatitis, STD, and TB Prevention, CDC.

U.S. clinical practice guidelines recommend directly observed therapy (DOT) as the standard of care for tuberculosis (TB) treatment (*1*). DOT, during which a health care worker observes a patient ingesting the TB medications, has typically been conducted in person. Video DOT (vDOT) uses video-enabled devices to facilitate remote interactions between patients and health care workers to promote medication adherence and clinical monitoring. Published systematic reviews, a published meta-analysis, and a literature search through 2022 demonstrate that vDOT is associated with a higher proportion of medication doses being observed and similar proportions of cases with treatment completion and microbiologic resolution when compared with in-person DOT (*2**–**5*). Based on this evidence, CDC has updated the recommendation for DOT during TB treatment to include vDOT as an equivalent alternative to in-person DOT. vDOT can assist health department TB programs meet the U.S. standard of care for patients undergoing TB treatment, while using resources efficiently.

## Background

The 2016 U.S. clinical practice guidelines for TB treatment recommend DOT as the standard of care (*1*). During DOT, a health care worker observes patients ingest their medications, monitors them for adverse events, and provides social support (e.g., personal connection, encouragement, advice, or assistance navigating challenges that occur with illness). Typically, DOT has involved meeting in person at a mutually agreed-upon location within the community or in a clinical setting; however, participation in DOT in person can be logistically challenging. Scheduling can interfere with patients’ employment, schooling, or other daily activities, and arranging transportation for DOT can be difficult. With community-based DOT, the daily arrival and departure of health care workers might also prompt unwelcome questions from neighbors or coworkers or result in the creation of stigma for the patient. Moreover, in-person DOT might not always be feasible during inclement weather, natural disasters, or a pandemic.

vDOT (also known as video DOT) allows persons undergoing TB treatment the opportunity to use video-enabled phones, tablets, or computers to remotely interact with health care workers in real time (synchronous) or through recorded videos (asynchronous). CDC reviewed published evidence on vDOT compared with in-person DOT for TB treatment adherence, completion, and microbiologic resolution to update the 2016 clinical practice guidelines (*1*). This update is for organizations and providers responsible for providing care for and monitoring treatment of persons with diagnosed TB in the United States and affiliated areas. Additional considerations, concerns, and limitations are available.*

## Methods

CDC developed these guidelines based on evidence presented by a systematic review and a meta-analysis that included studies published from the time the searched databases were initially available through January 2021 (*2*). An additional search of articles published during February 1, 2021–May 13, 2022, was conducted to identify subsequent studies that were not included in the systematic review and meta-analysis. The search of articles listed in PubMed, Embase, and Cochrane databases was conducted using the keywords “tuberculosis” and “directly observed therapy”; “directly observed treatment”; “video observed”; “video supported”; “adherence”; “treatment completion”; or “cell-,” “smart-,” or “tele-” “phone.” Studies were excluded if they did not report data for treatment adherence, treatment completion, or microbiologic testing; did not have a comparison group; or focused on the use of text message reminders or device-facilitated monitoring without video capability (e.g., medication containers with wireless sensors or ingestible sensors). Studies were also excluded if they compared vDOT with self-administered therapy or reported populations undergoing TB treatment in an inpatient, institutional, or medically supervised residential setting (e.g., a rehabilitation center). Two reviewers screened article abstracts for exclusion criteria and then independently documented participant demographics, DOT methods, doses scheduled for DOT, medication adherence, and treatment outcomes from retained articles that met inclusion criteria. Studies involving persons of any age, any sex, and from any upper-middle– to high-income country with a diagnosis (or suspected diagnosis) of TB, including pulmonary disease, extrapulmonary disease, and drug-resistant TB, undergoing treatment in an outpatient setting were included (*6*). The Methods Manual for Community Guide Systematic Reviews provided a framework for data collection from retained articles (*7*). Consistent with the evidence quality tools used in the published meta-analysis, retained articles were reviewed with the Revised Tool for Assessing Risk of Bias in Randomized Trials, the Newcastle-Ottawa Scale and Agency for Healthcare Research and Quality Standards (*2*,*6*–*9*). During May–September 2022, CDC reviewed the evidence and drafted recommendations. These recommendations were reviewed favorably by external TB subject matter experts and were presented for public comment during the December 2022 Advisory Council for the Elimination of Tuberculosis^†^ meeting. Comments supported the updated recommendations without further modifications.

## Rationale and Evidence

**Literature Review.** Two systematic reviews that assessed technology interventions for TB treatment were identified (*2*,*3*). The first review combined vDOT, text reminders, and medication monitoring boxes for comparison with in-person DOT (*3*); because of the combination of interventions assessed, this review was excluded. The second review, a meta-analysis comparing vDOT with in-person DOT, assessed treatment adherence, treatment completion, and microbiologic resolution (*2*). This published meta-analysis was used as supporting evidence and as the starting point for an updated literature search. The updated literature search yielded five articles published after the meta-analysis, two of which were retained as supporting evidence (*4*,*5*). Three articles were excluded for the following reasons: two did not include a comparison group (*10*,*11*), and one reported previously published data (*12*) included in the meta-analysis (*2*).

## Evidence Summary

**Treatment Adherence.** The meta-analysis (*2*), one randomized controlled trial (RCT) (*4*), and one prospective observational study (*5*) examined the proportion of medication doses observed by TB program staff members (Table). The meta-analysis defined treatment adherence as observation of ≥80% of prescribed doses. The RCT and observational study defined adherence as the observed proportion of total prescribed doses. The meta-analysis and observational study found higher adherence among patients on vDOT than among those receiving in-person DOT (78.8% versus 27.2%, and 68.4% versus 53.9%, respectively). The observational study focused on doses taken Monday through Friday (*5*). Per program practice, if a patient using vDOT missed a weekday dose and submitted additional videos on the weekend, these doses were included in the weekly adherence count. The RCT found that vDOT was as effective as in-person DOT at achieving observed doses (89.8% versus 87.2%) (*4*).

**TABLE:** Summary of evidence for the use of video directly observed therapy in the treatment of tuberculosis — United States, 2023

Publication	Study design	Setting and location	DOT modalities compared	Study population	Outcome	Definition	Descriptive result	Statistical measure	Conclusion
Truong CB, Tanni KA, Qian J.*	Systematic review and meta-analysis	TB program settings in Australia, China, Moldova, United Kingdom, and United States	Synchronous or asynchronous vDOT compared with community or clinic-based in-person DOT	Patients being treated for TB or LTBI for 4–9 mos	Adherence	Patient took ≥80% of prescribed doses	vDOT 360/457 (78.8%) patients: in-person DOT 106/390 (27.2%) patients	RR (95% CI) = 2.79 (2.26 to 3.45)	Better outcome with vDOT compared with in-person DOT
					Treatment completion	Patient did not prematurely stop treatment or was not lost to follow-up	vDOT 124/157 (79.0%) patients; in-person DOT 436/639 (68.2%) patients	RR (95% CI) = 1.33 (0.73 to 2.43)	vDOT and in-person DOT are equivalent
					Microbiologic resolution	Radiography and negative sputum smear in the last month of treatment and on one or more previous occasions among patients who were sputum smear positive at beginning of treatment	vDOT 304/327 (93.0%) patients; in-person DOT 289/329 (87.8%) patients	RR (95% CI) = 1.06 (1.01 to 1.11)	Better outcome with vDOT compared with in-person DOT
Perry A, Chitnis A, Chin A, et al.^†^	Prospective observational study	Urban TB program, Alameda County Public Health Department, California	Asynchronous vDOT compared with community-based in-person DOT	Patients receiving care for TB treatment during 2018–2020	Adherence	Proportion of total prescribed doses verified by observation with weekend and holiday self-administration^§^	vDOT 68.4% of doses; in-person DOT 53.9% of doses	p<0.001	Better outcome with vDOT compared with in-person DOT
					Treatment completion	Treatment completion and success were based on ingesting a set number of target doses	vDOT 96% of patients; in-person DOT 90% of patients	p = 0.326	vDOT and in-person DOT are equivalent
					Microbiologic resolution	Mean days to culture conversion among patients who were sputum smear positive at beginning of treatment	vDOT 48 days; in-person DOT 47 days	p = 0.843	vDOT and in-person DOT are equivalent
Burzynski J, Mangan JM, Lam CK, et al.^¶^	Randomized controlled trial	Urban TB program in four clinics, NYC DOHMH, New York	Synchronous and asynchronous vDOT compared with community and clinic-based in-person DOT	173 patients in 8-wk crossover periods	Adherence	Percentage of medication doses participants were observed to completely ingest	vDOT 89.8% of doses; in-person DOT 87.2% of doses**	Percentage difference^††^ (95% CI) = −2.6% (−4.8% to −0.3%)	vDOT and in-person DOT are equivalent (trial used a noninferiority design)

**Abbreviations**: DOT = directly observed therapy; LTBI = latent tuberculosis infection; MITT = modified intention to treat; NYC DOHMH = New York City Department of Health and Mental Hygiene; RR = risk ratio; TB = tuberculosis; vDOT = video directly observed therapy.

* https://doi.org/10.1016/j.amepre.2021.10.013

^†^ https://doi.org/10.5588/ijtld.21.0170

^§^ Study focused on doses taken Monday through Friday. Per program practice, if a patient using vDOT missed a weekday dose and submitted additional videos on the weekend, these were included in counts to confirm adherence for 5 of 7 days of the week. CDC notes this approach to quantifying treatment adherence could potentially bias results in favor of vDOT.

^¶^ This study did not evaluate treatment completion or microbiologic resolution. https://doi.org/10.1001/jamanetworkopen.2021.44210

** Results from the MITT analysis. Empirical, per-protocol, and per-protocol 85% analyses were also conducted and had noninferiority results consistent with those from the MITT analysis.

^††^ Calculated by subtracting the percentage of completed doses observed with electronic DOT from the percentage with in-person DOT.

**Treatment Completion.** The meta-analysis (*2*) defined completion of treatment as not prematurely stopping treatment or being lost to follow-up. The observational study (*5*) defined completion based on a set number of target doses (Table). Treatment completion was similar among patients receiving vDOT and in-person DOT (79.0% versus 68.2%, respectively, in the meta-analysis, and 96% versus 90%, respectively, in the observational study). The RCT did not evaluate treatment completion (*4*).

**Microbiologic Resolution**. The meta-analysis (*2*) and observational study (*5*) reported results for microbiologic resolution, the principal prognostic indicator for TB treatment response. The RCT did not evaluate microbiologic outcomes (*4*). Meta-analysis results were based on radiography and negative sputum smear test results by the last month of treatment and on at least one previous occasion. The observational study reported microbiologic resolution as the mean number of days to culture conversion (i.e., time between treatment start date and date of first negative culture result, after which no further positive culture results were obtained). Microbiologic resolution was similar between patients receiving vDOT and in-person DOT (93.0% versus 87.8%, respectively, in the meta-analysis, and a mean of 48 days versus 47 days, respectively, to culture conversion in the observational study).

## Updated Recommendation

Missed doses of medication or treatment interruptions can lead to suboptimal drug concentrations, acquired drug resistance, longer treatment times, TB treatment failure, and recurrence of TB disease. For these reasons, _CDC continues to recommend DOT as the standard of care for all persons prescribed TB treatment; however, based_ on the evidence summary, _this report updates the 2016 CDC U.S. clinical practice guidelines (_*_1_*_) to state that vDOT should be considered equivalent to in-person DOT._

## Considerations

Decisions regarding the use of vDOT or in-person DOT during TB treatment are best made when health care providers and patients work in partnership to discuss the potential benefits and drawbacks of both DOT approaches. Topics to address in shared decision-making discussions include the patient’s health care needs, social conditions, preferences, regular access to video-enabled devices and the Internet, insurance reimbursement (as applicable), confidentiality and privacy, as well as program capacities and provider preferences. For patients receiving injectable medications, experiencing circumstances that they and their providers decide would benefit from additional monitoring, or who are unable to use vDOT technology, in-person DOT is likely the better treatment option.

## Discussion

This update of CDC recommendations is based on evidence that vDOT is associated with a higher proportion of medication doses being observed and similar rates of TB treatment completion and microbiologic resolution when compared with in-person DOT. These data, combined with research that has demonstrated vDOT can conserve time and costs for patients and programs (*13*,*14*), improve patient satisfaction with DOT (*14*), and provide opportunities to monitor adherence when in-person DOT is not feasible (*5*), highlight the utility of vDOT to sustain patient care and treatment.

To date, few RCTs and cohort studies of vDOT have been conducted. Studies have been heterogenous with respect to video type (synchronous versus asynchronous) and location of in-person DOT (clinic versus community). In addition, published studies have been conducted in urban and suburban settings, with adults, and in locations with broad Internet availability. Thus, additional evaluation of vDOT implementation in more diverse settings and with diverse populations will address evidence gaps and expand the current knowledge base. Moreover, technology has evolved rapidly during the past decade, and this evolution will likely continue, adding to the evidence and further guiding best practices for the use of vDOT to support patients in their treatment adherence. CDC will continue to monitor relevant reports and update this guidance as necessary.

**Summary**

**What is already known about this topic?**

Directly observed therapy (DOT) for tuberculosis treatment involves observing a patient ingest medication, monitoring the patient for adverse events, and providing support for treatment completion. DOT has typically been conducted in person; however, scheduling in-person DOT can present logistical challenges.

**What is added by this report?**

Based on published evidence evaluating treatment adherence and completion and microbiologic resolution of disease, CDC recommends video DOT (vDOT) as equivalent to in-person DOT for persons undergoing treatment for diagnosed tuberculosis.

**What are the implications for public health practice?**

vDOT can assist health department tuberculosis programs meet the U.S. standard of care for patients undergoing tuberculosis treatment, while using resources efficiently.

## Footnotes

*
https://www.cdc.gov/tb/topic/treatment/vDOT.htm

†
https://www.cdc.gov/faca/committees/pdfs/acet/acet-minutes-20221213-14-508.pdf
